# Environmental, Health and Sociodemographic Determinants Related to Common Mental Disorders in Adults: A Spanish Country-Wide Population-Based Study (2006–2017)

**DOI:** 10.3390/jcm9072199

**Published:** 2020-07-12

**Authors:** Jesús Cebrino, Silvia Portero de la Cruz

**Affiliations:** 1 Department of Preventive Medicine and Public Health, Faculty of Medicine, University of Seville, Avda. Doctor Fedriani, S/N, 41009 Seville, Spain; 2 Department of Nursing, Pharmacology and Physiotherapy, Faculty of Medicine and Nursing, University of Córdoba, Avda. Menéndez Pidal, S/N, 14071 Córdoba, Spain; n92pocrs@uco.es

**Keywords:** anxiety disorders, depressive disorder, environment and public health, human, mental disorders, trends

## Abstract

Common mental disorders (CMD) represent a serious, growing public health concern, especially in women. The aims of this study were to report the prevalence of CMD among the adult population in Spain, to analyze the time trends from 2006 to 2017 and to explore the associations between CMD and gender, in relation to the perceived environmental and sociodemographic problems and clinical factors. A nationwide cross-sectional study was conducted including 48,505 participants aged 16 to 64 years old who had participated in the Spanish National Health Surveys in 2006, 2011/2012 and 2017. A logistic regression analysis was performed to identify the variables associated with CMD by gender. The prevalence of CMD was 20.4% in 2006, 20.8% in 2011/2012 and 16.9% in 2017 (*p* = 0.36). In women, the probability of having a CMD was higher in widowed or separated/divorced compared with single individuals and as the perception of distressing noise levels from outside the home increased. The probability of CMD was lower as the level of education increased in men. Foreigners and those with limitations due to health problems, chronic conditions and worse perceived health were more likely to suffer from a CMD in both women and men.

## 1. Introduction

Mental health is gaining recognition as one of the priority areas in health policies worldwide and has also been included in Sustainable Development Goals [1,2]. Nevertheless, most countries globally are exposed to multiple psychosocial stress factors nowadays [3]. For this reason, mental disorders are a high priority in healthcare as major contributors to the global burden of disability, especially in developing countries [4,5]. Estimates of the 12-month prevalence of mental disorders vary between 27.4% and 38.2% in the general European adult population [6,7], due to the inclusion of new disorders and of new European member states. In fact, European countries show that they suffer relatively more from mental health disorders (as a percentage of the total disease burden) than the rest of the world, although these may be significantly underdiagnosed in many countries [8,9,10].

Common mental disorders (CMD) refer especially to anxiety and mood disorders, which are highly prevalent in the global population (with a range from 3.6% to 19.8% for anxiety disorders [7,11,12,13] and from 5.4% to 7.8% for mood disorders [7,12,13]. Approximately one in five adults experience a CMD yearly and 29.2% at one point in their lifetime [12]. In Spain, the prevalence of CMD in adults ranges from 16% to 22% [14,15,16]. CMDs not only pose a threat to people’s quality of life, but they also pose serious challenges for health systems and entail considerable societal and financial costs [17,18]. Therefore, both researchers and policy-makers have identified decreasing the prevalence rates of CMD and disability costs as a key priority [7,19,20,21].

It seems that the factors associated with a high prevalence of CMD include sociodemographic and economic conditions, lifestyle habits and clinical variables [22,23]. Another characteristic which must be taken into account is the gender differences in relation to CMD, whereby women are more prone to experience CMD than men in most countries [12,24]. Nevertheless, these factors are not the only ones that influence the mental health of individuals [25], which also depends on the environmental context in which people are born, are raised and live their lives [26,27,28]. In short, there is a growing consensus that buildings, and the natural and social environments where people live, can affect mental health [28,29]. Conversely, certain environmental factors, such as green areas, could reduce stress and exert protective effects against mental disorders [30,31]. In contrast, air pollution and traffic noise have often been linked with poor mental health [32]. Moreover, stressful places, such as overcrowded urban areas, contribute to psychological stress [28]. In addition, Echeverría et al. [33] and Barnes et al. [34] analyzed people’s subjective perception about environmental problems and how it relates to mental health, and concluded that self-reports may be used as a good source of information even when they differ from objective measures. In fact, self-report perception of the environment is a more relevant measure of CMD than objectively-measured environmental factors.

Since the majority of studies assess environmental factors that could be related to CMDs jointly [35,36,37], making it impossible to know how each factor contributes to the presence or absence of CMD, the present study aims to investigate numerous different sociodemographic, clinical, lifestyle characteristics and environmental problems independently and simultaneously in a large, representative sample of Spanish population. The main objectives of the present study were to report the prevalence of CMD among the adult population in Spain, to analyze time trends from 2006 to 2017 and to explore the associations between CMD and gender in regard to both perceived environmental problems and sociodemographic, clinical and lifestyle factors.

## 2. Materials and Methods

### 2.1. Study Design

A quantitative, observational, nationwide, cross-sectional study carried out from December 2019 to April 2020.

### 2.2. Data Source and Study Population

Data were obtained from the personalized interviews of the Spanish National Health Survey (SNHS) 2006 [38], 2011/2012 [39] and 2017 [40]. The SNHSs are interview-based surveys of the non-institutionalized population (representativeness is ensured by assigning a weighting coefficient to each participant) carried out by the Ministry of Health, Consumer Affairs and Social Welfare in partnership with the National Institute of Statistics. The sampling design was multistage probabilistic, stratified by census areas (first stage), family homes (second stage) and individuals (third stage).

For the purpose of this work, we limited our study to people aged 16 to 64 years living in Spain. The initial sample consisted of 52,469 subjects, but due to a lack of data for some of the variables studied, 3964 (7.55%) individuals were excluded (SNHS 2006: 1775; SNHS 2011/2012: 1190 and SNHS 2017: 999) when the descriptive, bivariate and multivariate statistical analyses were carried out. Based on the sociodemographic, clinical, lifestyle and environmental variables and the presence of CMD, the subjects we excluded did not differ systematically from the rest of sample. Therefore, the study was finally based on 48,505 participants, as follows: 19,868 in SNHS 2006; 13,798 in SNHS 2011/2012; and 14,839 in SNHS 2017.

### 2.3. Variables

#### 2.3.1. Dependent Variable

The dependent variable was the presence of common mental disorders (CMD). This variable was evaluated using the General Health Questionnaire (GHQ-12), validated in Spain [41,42,43]. The GHQ-12 scored on a Likert-like scale from 0 (“more than usual”) to 3 (“much less than usual”). These answer categories were scored according to the original GHQ method [44], with the first two response options having a score of 0 and the last two having a score of 1, in other words, in bimodal fashion (0-0-1-1). The points were added up make to a global score ranging from 0 to 12 points. The GHQ-12 scoring method with a cut-off at ≥3 points was used, so that the final score is a dichotomized measurement of common mental disorders: absence of CMD (<3 points) and presence of CMD (≥3 points), the latter being used to indicate risk for psychological distress.

#### 2.3.2. Sociodemographic Variables

The independent variables, such as the sociodemographic variables, were: year of survey (2006, 2011/2012, 2017), gender (male, female), age group (16–24 years, 25–44 years, 45–64 years), marital status (single, married, widowed, separated or divorced), level of education (without studies, primary, secondary or professional training, university), nationality (Spanish, foreigner), size of town of residence (<10,000 inhabitants, 10,000–100,000 inhabitants, >100,000 inhabitants) and employment situation (employment, unemployment), using the International Labour Organization criteria [45]. Social class was assigned according to the categories proposed by the Spanish Society of Epidemiology [46]. This independent variable was classified into: Class I (directors and managers of companies with 10 or more employees and professionals normally qualified with university degrees), Class II (directors and managers of companies with less than 10 salaried employees and professionals normally qualified with university degrees and other technical support professionals). Athletes and artists), Class III (intermediate professions and self-employed workers), Class IV (supervisors and workers in skilled technical work), Class V (skilled workers in the primary sector and other semi-skilled workers), Class VI (unskilled workers). In this study, these six original classes were formed into three groups (upper class: Classes I and II, middle class: Classes III and IV, lower class: Classes V and VI).

#### 2.3.3. Lifestyle Behavior

Lifestyle behavior included physical activity during leisure time (Yes, I do physical activity/No, I do not do physical activity).

#### 2.3.4. Clinical Variables

The clinical variables were evaluated using a self-developed questionnaire proposed by the Spanish Ministry of Health, Consumer Affairs and Social Welfare [47,48,49] and included self-perceived health status (very good, good, fair, poor, very poor) and limitations due to a health problem for at least 6 months (limited, not limited). Another clinical characteristic was type(s) of chronic disease(s) as assessed by a physician. This variable was classified as number of chronic conditions (none, 1–2, ≥3). The chronic diseases included in the present study were: hypertension, myocardial infarction, angina pectoris/coronary disease, other heart diseases, varicose veins in the legs, osteoarthritis (excluding arthritis), chronic back pain (cervical), chronic back pain (lumbar), chronic allergy (such as rhinitis, conjunctivitis or allergic dermatitis, food allergy or other allergy [not including allergic asthma]), asthma (including allergic asthma), chronic bronchitis/emphysema/chronic obstructive pulmonary disease, diabetes, stomach ulcer/duodenum ulcer, urinary incontinence/problems controlling urine, high cholesterol, cataracts, chronic skin problems, chronic constipation, cirrhosis/liver dysfunction, depression, chronic anxiety, other mental problems, stroke (embolism, cerebral infarction, cerebral hemorrhage), migraine/frequent headaches, hemorrhoids, malignant tumors, osteoporosis, thyroid problems, kidney problems, prostate problems and menopausal problems.

#### 2.3.5. Social Support

To collect perceived personal social support information as an independent variable, the Duke-UNC-11 questionnaire [50], validated in Spain [51,52], was used. This scale includes 11 items which are scored on a Likert-like scale ranging from 1 (“Much less than I would like”) to 5 (“As much as I would like”). The overall perceived social support is obtained by adding the scores of the 11 items. The results range from 11 to 55 points. The final score is a dichotomized measurement of the perceived social support, classified into optimal support (≥33 points) and sup-optimal support (≤32 points).

#### 2.3.6. Perception of Environmental Problems

This variable was derived from the section “Problems the person has with the environment of their dwelling” [53,54,55]. This section includes the following problems: (i) noise from outside bothers you, (ii) bad smells coming from outside, (iii) drinking water is of poor quality, (iv) little street cleaning, (v) air pollution caused by a nearby industry, (vi) increased air contamination by other causes, (vii) lack of green areas, (viii) presence of animals that could be a nuisance (cats, dogs, pigeons, etc.)”. Each is problem scored on a Likert-like scale from 0 (“none”) to 2 (“a great deal”). In addition, the number of environmental problems perceived was collected. This number was obtained by adding together all the environmental factors.

### 2.4. Ethical Aspects

The data obtained from these surveys are available on the National Institute of Statistics and Ministry of Health, Consumer Affairs and Social Welfare websites [38,39,40] in the form of anonymized microdata; no special authorizations are therefore required for their use. According to the SNHS methodology, the microdata files are anonymous and are available to the public. In accordance with Spanish legislation, when secondary data are used, there is no need for approval from an Ethics Committee. The research data are available as a Supplementary File.

### 2.5. Statistical Analysis

A descriptive analysis was performed by calculating the counts and percentages for the categorical variables and the continuous variables by calculating the arithmetic mean and standard deviation (SD). Differences in the prevalence of CMD within the three time points (2006, 2011/2012 and 2017) were compared using the Chi-square test for contingency tables or Fisher’s exact test if the number of expected frequencies was greater than 5. Linear regression models were used to identify statistically significant trends in (a) the prevalence of CMD, (b) sociodemographic, clinical, lifestyle characteristics and (c) the perception of environmental problems in the period of 2006–2017. The regression coefficient and the coefficient of determination (R^2^) were calculated to assess the direction, average magnitude of the change and performance of the models. In addition, a logistic regression was performed to identify the variables associated with CMD by gender. All variables with a significant association in the bivariate analysis were included in the multivariate analysis. Crude and adjusted odds ratios (ORs) were obtained for all sociodemographic, clinical and lifestyle habits variables and perception of environmental problems, with 95% confidence intervals (95% CI). The Wald statistic was used to exclude one by one from the model any variables with a *p* ≥ 0.15 (backward methodical selection procedure). All the hypothesis contrasts were bilateral and in all the statistical tests, those with a 95% CI (*p* < 0.05) were considered significant values. The statistical analysis was carried out using IBM SPSS Statistics version 25 (IBM Corp, Armonk, NY, USA).

## 3. Results

### 3.1. Sociodemographic, Clinical and Lifestyle Habits Variables

The study population included 48,505 individuals, of whom 26,462 were women (54.6%) and 22,043 were men (45.4%) between 16 and 64 years old. The most frequent sociodemographic, clinical and lifestyle characteristics of the participants were that they were married (57.7%), Spanish (93.7%) and employed (63.3%), belonged to middle-class families (41.4%), had secondary or professional training (57%), lived in towns with a population of over 100,000 inhabitants (40.4%), had a good self-perceived health status (53.6%), had one or two chronic conditions (36.9%), had not reported any limitations due to a health problem for at least 6 months (81.2%), did physical activity during their leisure time (60.7%) and had normal social support (97%).

### 3.2. Perception of Environmental Problems

**Figure 1:**
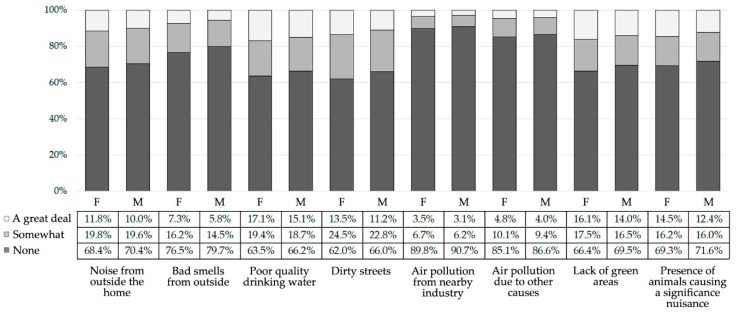
Perception of environmental problems of people aged from 16 to 64 years in Spain (N = 48,505) (2006–2017). F: female; M: male.

According to Figure 1, the vast majority of participants had not perceived environmental problems. Furthermore, within those who did perceive them, the most common environmental problems were distressing levels of noise from outside the home (a great deal: 11.8%, somewhat: 19.8% in women; a great deal: 10.0%, somewhat: 19.6% in men), poor quality drinking water (a great deal: 17.1%, somewhat: 19.4% in women; a great deal: 15.1%, somewhat: 18.7% in men), and dirty streets (a great deal: 13.5%, somewhat: 24.5% in women; a great deal: 11.2%, somewhat: 22.8% in men).

### 3.3. Common Mental Disorders

The overall prevalence of CMD was 19.4% (23.1% in women and 15.0% in men). Over the different years of the study, the prevalence of CMD was 20.4% in 2006, 20.8% in 2011/2012 and 16.9% in 2017 (β = −0.32, R^2^ = 0.72, *p* = 0.36). By gender, the prevalence of CMD in women was 24.5% in 2006, 24.1% in 2011/2012 and 20.1% in 2017 (β = −0.41, R^2^ = 0.87, *p* = 0.24) and in men was 14.4% in 2006, 17.4% in 2011/2012 and 13.5% in 2017 (β = −0.09, R^2^ = 0.06, *p* = 0.84).

### 3.4. Evolution of Sociodemographic, Clinical and Lifestyle Habits Variables and Perception of Environmental Problems

As regards the sociodemographic, clinical and lifestyle habits variables of the sample according to the year of the survey (Table 1), a decrease in the number of people who were between 25 and 44 years old was seen (2006: 48.3%, 2011/2012: 45.3%, 2017: 40.9% (β = −0.68, R^2^ = 0.92, *p* = 0.03)). There was also an increase in separated or divorced people [2006: 6.3%, 2011/2012: 7.6%, 2017: 9.0% (β = 0.24, R^2^ = 0.99, *p* = 0.02)]. The overall number of environmental problems perceived was 2.10 (SD ± 1.86). In addition, it should be noted that there was an increase of participants who did not perceive any noise from outside the home (2006: 67%, 2011/2012: 69.6%, 2017: 72.1% (β = 0.46, R^2^ = 0.99, *p* = 0.04)).

### 3.5. Association between Sociodemographic, Clinical and Lifestyle Habits Variables, Perception of Environmental Problems and Common Mental Disorders

In women, the adjusted logistic regression model showed that the probability of having CMD was higher in those who were widowed (OR = 1.10, 95%CI 1.08–1.25), separated or divorced (OR = 1.16 95%CI 1.03–1.31), foreigners (OR = 1.51, 95%CI 1.33–1.71), unemployed (OR = 1.25, 95%CI 1.17–1.34), did no physical activity during their leisure time (OR = 1.31, 95%CI 1.23–1.40) and perceived a low social support (OR = 3.55, 95%CI 3.01–4.19). In addition, the probability of having CMD was greater as the distress caused by noise from outside the home increased (somewhat: OR = 1.05, 95%CI 1.02–1.10; a great deal: OR = 1.17, 95%CI 1.05–1.30), as perceived health was worse (good: OR = 1.49, 95%CI 1.34–1.67; fair: OR = 3.10, 95%CI 2.75–3.51; poor: OR = 5.76, 95%CI 4.89–6.79; very poor: OR = 10.93, 95%CI 8.46–14.13) and the number of chronic conditions increased (1-2: OR = 1.43, 95%CI 1.30–1.57; ≥3: OR = 2.20, 95%CI 1.99–2.42). Moreover, for each increase in a perceived environmental problem, the probability of CMD increased 1.29 times (OR = 1.29, 95%CI 1.17–1.43). By contrast, the probability of CMD decreased in women who were not limited by a health problem (OR = 0.56, 95%CI 0.52–0.61).

In men, the probability of having CMD increased in those who were foreigners (OR = 1.56, 95%CI 1.33–1.83), unemployed (OR = 1.93, 95%CI 1.77–2.10), did no physical activity during their leisure time (OR = 1.35, 95%CI 1.24–1.46) and perceived a low social support (OR = 3.15, 95%CI 2.63–3.76). Furthermore, the probability of having CMD was greater as perceived health was worse (good: OR = 1.65, 95%IC 1.44–1.87; fair: OR = 3.17, 95%CI 2.71–3.71; poor: OR = 6.23, 95%CI 5.06–7.67; very poor: OR = 8.88, 95%CI 6.42–12.28) and the number of chronic conditions increased (1-2: OR = 1.28, 95%CI 1.16–1.43); ≥3: OR = 1.70, 95%CI 1.51–1.92). In addition, for each increase in a perceived environmental problem, the odds of CMD increased 1.18 times (OR = 1.18, 95%CI 1.07–1.32). On the contrary, the probability of having CMD decreased as the level of education increased (primary: OR = 0.96, 95%CI 0.85–0.98; secondary or professional training: OR = 0.80, 95%CI 0.70–0.93, university: OR: 0.53, 95%CI 0.43–0.66) and in those who were not limited by a health problem (OR = 0.48, 95%CI 0.43–0.53) (Table 2). 

## 4. Discussion

### 4.1. Main Findings

The results of this study based on a large and representative adult population between 16–64 years old in Spain are unique in showing the relationship between perception of environmental problems, sociodemographic, clinical and lifestyle characteristics and CMD from 2006 to 2017, taking into account potential gender differences.

The overall prevalence of CMD was higher among women (23.10%) than men (15.03%). These percentages were similar when compared to previous studies conducted among the general and non-institutionalized population living in Spain, which ranged from 18% to 30% in women and 9% to 18% in men [14,15,16,56,57,58,59,60,61]. Although the causes of these differences are still not understood completely [62], the variation observed may be due to multiple potential factors, such as hormone mechanisms [63], gender-based violence [64], traditional gender roles [65], discrimination in the workplace or during professional careers [66], low social support [67] and more likelihood of them being willing to talk about their feelings and to seek help from healthcare services [68,69]. Particular attention should be given to the socio-economic situation in the European region, which has deteriorated markedly since 2007–2008 [70]. For this reason, the economic crisis had a particularly adverse impact in Spain, negatively affecting mental health [71,72,73]. This confirms our results which showed higher percentages of CMD in 2006 and 2011–2012. Nonetheless, from 2015, as the Spanish economy improved [74], the consequences of the previous recession were felt not the same by everybody [75]. In other words, unemployed individuals and people without studies suffered a more detrimental effect on their mental health from the economic crisis [76,77,78]. For that reason, the low percentage of CMD obtained in 2017 compared to previous years may be explained by the higher percentages of people who had a job and a high level of education in the same year. During the economic crisis in Spain, workers who had lost their jobs decided to return to education [79], perhaps because some men held traditional views about their role, as the family bread winner [60,77]. This corroborates the results obtained, in which the probability of having CMD decreased as the level of education increased in men. Among the higher educated, more rational coping strategies allow the individual to raise questions, solve problems and progress in their lives [80].

It was found that people with a low level of perceived social support had an increased probability of suffering from CMD, in line with other studies in Spain [15,58,61,81,82]. Perceived social support refers to people’s beliefs about how much support is potentially available from their relationships and social contacts and about the quality of this support [83,84]. Its beneficial effects for health are either direct, allowing the individual to enjoy a sense of general wellness, or indirect, as a stress-buffering process [85,86].

Foreigners had an increased likelihood of suffering from CMD. There may be many reasons for this: the emotional burden of the migratory process, unfavorable living conditions, general ignorance of the country of residence, a precarious socioeconomic situation, the difficulty of learning and communicating in a new language, family upheaval or the strong cultural shock they experience [87,88,89].

In the present study, in both genders, the likelihood of suffering from CMD was higher when the self-perceived health status was worse, as concluded by Henares-Montiel et al. [16]. Moreover, low self-perceived health can lead to problems of physical functional status and concurrent problems with certain chronic diseases [90,91]. Similarly, our results showed that adults without limitations due to a health problem had less probability of having CMD and those with at least one chronic condition were more likely to suffer CMD, as shown in other studies [58,61,81]. Limitations due to a health problem are a serious source of strain and have a direct influence on CMD [92]. On the other hand, there is a broad consensus in the scientific literature regarding the association between the presence of some chronic disease and poor mental health [93,94,95]. There are many reasons for this, including chronic pain, restrictions on social life or somatic discomfort, which are common consequences of chronic diseases that increase the risk of mental problems [96].

Regarding marital status, women who were widowed or separated/divorced had more probability of suffering from CMD, as is also reflected in the extensive body of literature [97,98,99]. Marital disruption (separation/divorce or widowhood) can be an extremely stressful event [100] and may result in poorer mental health [101,102,103]. In this context, the level of psychological anxiety among divorcees and widowers is on average 36% higher compared to people whose marital status has not changed [104]. Gender differences in the impact of separation or divorce are driven by women’s poorer prospects of remarriage. Given that the decline in the probability of remarriage after separation or divorce with increasing age is steeper and starts earlier for women than for men, this potential source of gender differences is relatively pronounced among separated and divorced individuals [105]. On the other hand, the results of research show that the worse mental health status for widowed women is caused by the decline of socio-economic status after a spouse’s death [104].

It was found that the probability of suffering from CMD was higher in women who perceived a great deal of or somewhat noise from outside the home than in men. In general, women show higher levels of impaired mental health when exposed to all noise sources and annoyance levels than men [106]. Nevertheless, no gender differences have been found in relation to noise sensitivity [107], although some studies have shown that it was higher in women [108,109,110]. In fact, our results may be explained by attitudes towards noise: women are more attentive to the risk of hearing loss, especially when exposed to loud noise, and are four times more prone to use protective devices [108]. In this study, distressing levels of noise from outside the home were a common environmental problem perceived by participants. It has been hypothesized that the quality of a person’s environment (clean, comfortable and safe) has been linked to their levels of psychological distress, which entails a reduction of CMD [111]. Although there are disparities in the results [112], distressing levels of noise from outside the home are associated with impaired mental health [113,114,115]. Besides the damage to hearing caused by loud sounds, noise can lead to extra-auditory effects, such as stress reactions, or can interfere with communication or concentration [116]. Noise from outside the home is usually caused by nearby traffic, while distressing noise inside the home is usually caused by home appliances, heating, ventilation and air conditioning systems [110,117]. This noise impacts on sleep patterns, therefore adversely affecting health [118]. In fact, several studies have documented the relationship between distressing noise and sleep disturbance [119,120,121,122] and the latter is considered a risk factor for CMD [123]. Moreover, long-term exposure to distressing noise at home could also lead to several health problems such as mental illness [117].

### 4.2. Strengths and Limitations

Some limitations to this study need to be recognized. Firstly, due to the cross-sectional design, it was not possible to assign causality between the sociodemographic, clinical and lifestyle characteristics, perception of environmental problems and CMD. Secondly, it should be noted that our indicators of mental health were constructed from self-reported survey questions, which can be affected by memory and/or social desirability bias. Nevertheless, the recall period is the past 12 months, which is relatively short for the dependent variables in GHQ-12. Thirdly, all participants were living at home; we do not possess data on institutionalized populations. Furthermore, it should be noted that people aged over 65 years were not included in this study, and therefore, the sample was not representative of all Spanish adults. On the other hand, one strength of our study is that since the data were derived from a national survey, they were obtained using a carefully planned methodology, including sampling, well-designed forms, preparation of the survey participants, supervision of the survey and filtering of the data, all of which guarantee a representative sample of the population between 16–64 years old and lead to a greater understanding of this problem in today’s society.

In this study, we identified vulnerable groups, such as unemployed people, foreigners or those who were limited due to a health problem. Nevertheless, longitudinal studies are needed to determine the best ways to reduce and prevent CMD in these groups. Finally, further prospective studies using experimental methods are needed to study the effects of distressing noise level and their consequences on the mental health of the population, for example, studies using sound meters to measure noise from outside the home.

### 4.3. Implications for Research and Practice

This large, representative sample of the Spanish population between 16 and 64 years old enabled us to investigate a considerable number of associations with factors from different domains simultaneously. Thus, this research provides valuable insights which will be useful for conducting future studies. Our findings show that the prevalence of CMD in non-institutionalized people in Spain from 2006 to 2017 was higher among women than in men. Furthermore, the overall prevalence of CMD was lower in 2017 compared to 2006 and 2011–2012, which represents a 4% decrease. Even so, further studies are needed to try to explain these gender differences in mental health in order to reduce them and promote equality. Our results show that foreigners, those with limitations due to health problems or chronic conditions and unemployed individuals were more likely to suffer from CMD in both women and men. These findings suggest that mental health policies should focus on vulnerable groups in Spain and apply health measures to prevent or reduce mental disorders. For example, work- or school-based mental health prevention programs could be implemented to promote physical activity and healthy nutrition [1]. Moreover, it would be desirable to offer training programs for unemployed people on coping strategies to help to reduce stress while job hunting [124,125]. It is equally important to pay sufficient attention to social support for people with common mental disorders, because learning about its beneficial effects may help these people to seek support from the healthcare services [126]. In fact, social activities are often overlooked in health consultations, despite the recent growing demand to investigate social factors in mental health [127]. In addition, effective plans to promote social support in policy guidance are required [128]. Finally, our results confirm the negative impact of noise from outside the home on women’s mental health. It is necessary to adopt political and social measures to try to reduce distressing noise levels, for example, creating more green areas, which play an important role in reducing noise and contribute to mental health well-being [129].

## 5. Conclusions

The overall prevalence of CMD in the adult population living in Spain is 19.43%, with a decrease in CMD in this population from 2006 to 2017. The probability of having CMD decreases as the level of education increases in men. In women, the probability of having CMD is higher in widowed and separated/divorced women compared with single participants and lower as the perception of distressing noise levels from outside the home decreases. In both groups, there is a clear increasing gradient in the probability of having CMD in adult foreigners and in those with some sort of limitation due to a health problem, and also when the self-perceived health is worse and the number of chronic conditions increases. By contrast, the likelihood of suffering from CMD decreases in employed people, in those who do physical activity during their leisure time and in those who perceive a normal level of social support.

## Figures and Tables

**Table 1:** Trends for sociodemographic, clinical, lifestyle and environmental variables of people aged from 16 to 64 years in Spain (N = 48,505) (2006–2017).

Variables	2006 n = 19,868 (%)	2011/2012 n = 13,798 (%)	2017 n = 14,839 (%)	*β*	R^2^	*p* for Trend
Gender						
Female	11,788 (59.3)	7014 (50.8)	7660 (51.6)	−0.67	0.62	0.42
Male	8080 (40.7)	6784 (49.2)	7179 (48.4)	0.67	0.62	0.42
Age group						
16–24 years	1979 (10.0)	1354 (9.8)	1294 (8.7)	−0.12	0.88	0.23
25–44 years	9602 (48.3)	6256 (45.3)	6072 (40.9)	−0.68	0.92	0.03
45–64 years	8287 (41.7)	6188 (44.9)	7473 (50.4)	0.79	0.97	0.07
Marital status						
Single	6079 (30.6)	4770 (34.6)	4694 (31.6)	0.07	0.04	0.87
Married	11,914 (60.0)	7607 (55.1)	8455 (57.0)	−0.25	0.32	0.61
Widowed	611 (3.1)	367 (2.7)	357 (2.4)	−0.06	0.96	0.12
Separated or divorced	1264 (6.3)	1054 (7.6)	1333 (9.0)	0.24	0.99	0.02
Social class						
Upper class	4235 (21.3)	2795 (20.3)	2972 (20.0)	−0.11	0.84	0.26
Middle class	10,363 (52.2)	4753 (34.4)	4964 (33.5)	−1.65	0.75	0.34
Lower class	5270 (26.5)	6250 (45.3)	6903 (46.5)	1.76	0.75	0.33
Level of education						
Without studies	1072 (5.4)	648 (4.7)	516 (3.5)	−0.18	0.98	0.07
Primary	6007 (30.2)	1155 (8.4)	1645 (11.1)	−1.67	0.59	0.44
Secondary or PT	9030 (45.4)	9355 (67.8)	9259 (62.4)	1.46	0.48	0.52
University	3759 (19)	2640 (19.1)	3419 (23.0)	0.38	0.83	0.27
Nationality						
Spanish	18,085 (91.0)	12,676 (92.0)	14,678 (99.0)	0.73	0.87	0.24
Foreigner	1783 (9.0)	1122 (8.0)	161 (1.0)	−0.73	0.87	0.24
Size of town of residence						
<10,000 inhab.	4965 (25.0)	3021 (21.9)	3215 (21.7)	−0.29	0.76	0.33
10,000–100,000 inhab.	7184 (36.2)	5013 (36.3)	5535 (37.3)	0.11	0.89	0.21
>100,000 inhab.	7719 (38.8)	5764 (41.8)	6089 (41.0)	0.19	0.46	0.52
Employment situation						
Employment	12,133 (61.1)	9064 (65.7)	9504 (64.0)	0.25	0.35	0.59
Unemployment	7735 (38.9)	4734 (34.3)	5335 (36.0)	−0.25	0.35	0.59
Self-perceived health status						
Very good	3446 (17.3)	3108 (22.5)	3461 (23.3)	0.53	0.81	0.29
Good	10,620 (53.5)	7601 (55.1)	7793 (52.5)	−0.10	0.16	0.73
Fair	4392 (22.1)	2375 (17.2)	2716 (18.3)	−0.33	0.50	0.50
Poor	1047 (5.3)	577 (4.2)	681 (4.6)	−0.06	0.33	0.61
Very poor	363 (1.8)	137 (1.0)	188 (1.3)	−0.05	0.38	0.58
Number of chronic conditions						
None	6284 (31.6)	5151 (37.3)	5539 (37.3)	0.50	0.70	0.37
1–2	7446 (37.4)	5073 (36.8)	5363 (36.2)	−0.12	0.99	0.06
≥3	6138 (31.0)	3574 (25.9)	3937 (26.5)	−0.38	0.59	0.44
Limitations due to a health problem for at least 6 months						
Not limited	15,879 (79.9)	11,675 (84.6)	11,852 (79.9)	−0.03	0.004	0.96
Limited	3989 (20.1)	2123 (15.4)	2987 (20.1)	0.03	0.004	0.96
Physical activity during leisure time						
No, I do not physical activity	7941 (40.0)	5790 (42.0)	5326 (35.9)	−0.39	0.49	0.51
Yes, I do physical activity	11,927 (60.0)	8008 (58.0)	9513 (64.1)	0.39	0.49	0.51
Perceived personal social support						
Low social support	557 (2.8)	402 (2.9)	518 (3.5)	0.06	0.90	0.21
Normal social support	19,311 (97.2)	13,396 (97.1)	14,321 (96.5)	−0.06	0.90	0.21
GHQ-12 score						
Absence of common mental disorders	15,817 (79.6)	10,931 (79.2)	12,331 (83.1)	0.33	0.72	0.36
Presence of common mental disorders	4051 (20.4)	2867 (20.8)	2508 (16.9)	−0.33	0.72	0.36
Noise from outside the home						
A great deal	2513 (12.6)	1441 (10.4)	1377 (9.3)	−0.29	0.94	0.15
Somewhat	4048 (20.4)	2756 (20.0)	2766 (18.6)	−0.16	0.93	0.16
None	13,307 (67.0)	9601 (69.6)	10,696 (72.1)	0.46	0.99	0.04
Bad smells from outside						
A great deal	1644 (8.3)	747 (5.4)	816 (5.5)	−0.24	0.67	0.38
Somewhat	3327 (16.7)	2005 (14.5)	2150 (14.5)	−0.19	0.70	0.37
None	14,897 (75.0)	11,046 (80.1)	11,873 (80.0)	0.44	0.69	0.38
Poor quality drinking water						
A great deal	3831 (19.3)	1717 (12.4)	2323 (15.6)	−0.31	0.24	0.67
Somewhat	3889 (19.6)	2387 (17.3)	2961 (20.0)	0.05	0.03	0.88
None	12,148 (61.1)	9694 (70.3)	9555 (64.4)	0.26	0.09	0.80
Dirty streets						
A great deal	3023 (15.2)	1193 (8.7)	1829 (12.3)	−0.23	0.15	0.73
Somewhat	5246 (26.4)	2859 (20.7)	3413 (23.0)	−0.28	0.30	0.63
None	11,599 (58.4)	9746 (70.6)	9597 (64.7)	0.52	0.22	0.69
Air pollution from nearby industry						
A great deal	923 (4.6)	337 (2.4)	367 (2.5)	−0.18	0.66	0.39
Somewhat	1432 (7.2)	875 (6.4)	829 (5.6)	−0.14	0.99	0.03
None	17,513 (88.2)	12,586 (91.2)	13,643 (91.9)	0.33	0.85	0.25
Air pollution due to other causes						
A great deal	1055 (5.3)	558 (4.1)	543 (3.7)	−0.14	0.89	0.21
Somewhat	2174 (10.9)	1246 (9.0)	1334 (9.0)	−0.16	0.70	0.36
None	16,639 (83.8)	11,994 (86.9)	12,962 (87.3)	0.31	0.79	0.30
Lack of green areas						
A great deal	4191 (21.1)	1441 (10.4)	1722 (11.6)	−0.83	0.60	0.43
Somewhat	3882 (19.5)	2177 (15.8)	2205 (14.9)	−0.41	0.85	0.25
None	11,795 (59.4)	10,180 (73.8)	10,912 (73.5)	1.23	0.69	0.38
Presence of animals causing a significance nuisance						
A great deal	2961 (14.9)	1549 (11.2)	2055 (13.8)	−0.08	0.05	0.84
Somewhat	3094 (15.6)	2213 (16.0)	2516 (17.0)	0.13	0.96	0.12
None	13,813 (69.5)	10,036 (72.8)	10,268 (69.2)	−0.05	0.02	0.92
Overall number of environmental problems perceived (Median ± Standard Deviation)	2.4 (1.9)	1.9 (1.9)	2.0 (1.8)	−0.03	0.52	0.49

PT: Professional Training; inhab.: inhabitants; GHQ-12: 12-item General Health Questionnaire.

**Table 2:** Association between common mental disorders and sociodemographic, clinical and lifestyle habits variables and perception of environmental problems by gender of adults aged from 16 to 64 years in Spain (N = 48505) (2006–2017).

Total (CMD)	Female (N = 26,462)						Male (N = 22,043)			
	*n* (%)	OR (95% CI)	*p*-Value	ORa (95% CI)	*p*-Value	*n* (%)	OR (95% CI)	*p*-Value	ORa (95% CI)	*p*-Value
	6114 (23.1)					3312 (15.03)				
Years of the surveys										
2006	2891 (24.5)	Reference	<0.01	Reference	<0.001	1160 (14.4)	Reference	<0.001	Reference	<0.001
2011/2012	1687 (24.1)	0.89 (0.88–1.11)		0.91 (0.87–1.15)		1180 (17.4)	1.26 (1.15–1.37)		1.52 (1.37–1.68)	
2017	1536 (20.1)	0.77 (0.72–0.83)		0.89 (0.83–0.97)		972 (13.5)	0.93 (0.85–1.02)		0.94 (0.85–1.04)	
Age group			<0.001					<0.001		
16–24 years	451 (19.2)	Reference				246 (10.8)	Reference			
25–44 years	2555 (21.3)	1.14 (1.02–1.28)				1441 (14.5)	1.40 (1.22–1.62)			
45–64 years	3108 (25.6)	1.45 (1.30–1.62)				1625 (16.5)	1.64 (1.42–1.89)			
Marital status										
Single	1560 (21.2)	Reference		Reference		1258 (15.4)	Reference			
Married	3419 (21.8)	1.03 (0.97–1.11)	0.34	0.75 (0.69–0.81)	0.21	1687 (13.8)	0.88 (0.81–0.95)	0.001		
Widowed	396 (36.2)	2.11 (1.84–2.41)	<0.001	1.10 (1.08–1.25)	<0.001	56 (23.2)	1.67 (1.23–2.26)	0.001		
Separated or divorced	739 (32.2)	1.76 (1.59–1.96)	<0.001	1.16 (1.03–1.31)	<0.001	311 (23.0)	1.64 (1.43–1.89)	<0.001		
Social class										
Upper class	955 (17.3)	Reference	<0.001			570 (12.7)	Reference	<0.001		
Middle class	2469 (22.8)	1.41 (1.30–1.53)				1321 (14.3)	1.15 (1.03–1.27)			
Lower class	2690 (26.7)	1.74 (1.60–1.89)				1421 (17.0)	1.41 (1.27–1.56)			
Level of education										
Without studies	485 (37.1)	Reference	<0.001			168 (18.1)	Reference	<0.001	Reference	<0.001
Primary	1335 (26.4)	0.61 (0.53–0.69)				642 (17.1)	0.94 (0.78–1.13)		0.96 (0.85–0.98)	
Secondary or PT	3344 (23.4)	0.52 (0.46–0.58)				2016 (15.1)	0.81 (0.68–0.96)		0.80 (0.70–0.93)	
University	950 (16.5)	0.33 (0.29–0.38)				486 (12.0)	0.62 (0.51–0.75)		0.53 (0.43–0.66)	
Nationality										
Spanish	5636 (22.7)	Reference	<0.001	Reference		3068 (14.9)	Reference	<0.01	Reference	<0.001
Foreigner	478 (28.5)	1.35 (1.21–1.51)		1.51 (1.33–1.71)	<0.001	244 (17.6)	1.23 (1.06–1.41)		1.56 (1.33–1.83)	
Size of town of residence										
<10000 inhab.	1269 (21.6)	Reference	<0.001			742 (13.9)	Reference	<0.001		
10000–100000 inhab.	2315 (23.7)	1.12 (1.04–1.22)				1269 (15.9)	1.17 (1.07–1.30)			
>100000 inhab.	2530 (23.4)	1.10 (1.02–1.19)				1301 (14.9)	1.08 (1.03–1.22)			
Employment situation										
Employment	3293 (28.4)	Reference	<0.001	Reference		1761 (11.1)	Reference	<0.001	Reference	<0.001
Unemployment	2821 (19.0)	1.69 (1.59–1.79)		1.25 (1.17–1.34)	<0.001	1551 (25.1)	2.68 (2.48–2.89)		1.93 (1.77–2.10)	
Self-perceived health status										
Very good	468 (9.3)	Reference	<0.001	Reference	<0.001	301 (6.0)	Reference	<0.001	Reference	<0.001
Good	2148 (15.7)	1.82 (1.63–2.02)		1.49 (1.34–1.67)		1367 (11.1)	1.94 (1.71–2.21)		1.65 (1.44–1.87)	
Fair	2277 (38.6)	6.13 (5.50–6.84)		3.10 (2.75–3.51)		1016 (28.3)	6.15 (5.40–7.05)		3.17 (2.71–3.71)	
Poor	873 (62.0)	15.87 (13.75–18.32)		5.76 (4.89–6.79)		474 (52.9)	17.49 (14.67–20.84)		6.23 (5.06–7.67)	
Very poor	348 (76.8)	32.29 (25.45–40.97)		10.93 (8.46–14.13)		154 (65.5)	29.60 (22.08–39.68)		8.88 (6.42–12.28)	
Number of chronic conditions										
None	858 (10.8)	Reference	<0.001	Reference	<0.001	772 (8.5)	Reference	<0.001	Reference	<0.001
1–2	1746 (18.6)	1.89 (1.73–2.06)		1.43 (1.30–1.57)		1202 (14.1)	1.77 (1.60–1.94)		1.28 (1.16–1.43)	
≥ 3	3510 (38.2)	5.09 (4.69–5.53)		2.20 (1.99–2.42)		1338 (29.9)	4.59 (4.16–5.06)		1.70 (1.51–1.92)	
Limitations due to a health problem for at least 6 months										
Not limited	3524 (16.8)	0.23 (0.21–0.24)	<0.001	0.56 (0.52–0.61)	<0.001	1967 (10.7)	0.20 (0.19–0.22)	<0.001	0.48 (0.43–0.53)	<0.001
Limited	2590 (47.2)	Reference		Reference		1345 (37.2)	Reference		Reference	
Physical activity during leisure time										
No, I do not physical activity	2949 (26.9)	1.43 (1.35–1.52)	<0.001	1.31 (1.23–1.40)	<0.001	1518 (18.8)	1.56 (1.45–1.69)	<0.001	1.35 (1.24–1.46)	<0.001
Yes, I do physical activity	3165 (20.4)	Reference		Reference		1794 (12.9)	Reference		Reference	
Perceived personal social support										
Low social support	470 (59.8)	5.28 (4.56–6.11)	<0.001	3.55 (3.01–4.19)	<0.001	289 (41.8)	4.36 (3.73–5.10)	<0.001	3.15 (2.63–3.76)	<0.001
Normal social support	5644 (22.0)	Reference		Reference		3023 (14.2)	Reference		Reference	
Noise from outside the home										
A great deal	1014 (32.6)	1.84 (1.69–1.99)	<0.001	1.17 (1.05–1.30)	<0.001	484 (21.8)	1.79 (1.60–2.01)	<0.001		
Somewhat	1335 (25.4)	1.30 (1.21–1.40)		1.05 (1.02–1.10)		734 (17.0)	1.31 (1.20–1.44)			
None	3765 (20.8)	Reference		Reference		2094 (13.5)	Reference			
Bad smells from outside										
A great deal	655 (33.9)	1.90 (1.72–2.10)	<0.001			276 (21.6)	1.69 (1.47–1.95)	<0.001		
Somewhat	1157 (27.0)	1.37 (1.27–1.48)				572 (17.9)	1.34 (1.21–1.48)			
None	4302 (21.2)	Reference				2464 (14.0)	Reference			
Poor quality drinking water										
A great deal	1278 (28.2)	1.50 (1.39–1.62)	<0.001			624 (18.7)	1.41 (1.28–1.56)	<0.001		
Somewhat	1350 (26.3)	1.37 (1.27–1.47)				643 (15.6)	1.14 (1.03–1.25)			
None	3486 (20.7)	Reference				2045 (14.0)	Reference			
Dirty streets										
A great deal	1077 (30.1)	1.65 (1.52–1.79)	<0.001			465 (18.8)	1.42 (1.27–1.59)	<0.001		
Somewhat	1635 (25.2)	1.29 (1.20–1.38)				810 (16.1)	1.18 (1.08–1.29)			
None	3402 (20.8)	Reference				2037 (14.0)	Reference			
Air pollution from nearby industry										
A great deal	276 (29.6)	1.44 (1.25–1.67)	<0.001			141 (20.3)	1.48 (1.22–1.79)	<0.001		
Somewhat	484 (27.3)	1.29 (1.16–1.44)				232 (17.0)	1.19 (1.03–1.38)			
None	5354 (22.5)	Reference				2939 (14.7)	Reference			
Air pollution due to other causes										
A great deal	412 (32.6)	1.71 (1.51–1.93)	<0.001			203 (22.7)	1.77 (1.50–2.08)	<0.001		
Somewhat	727 (27.1)	1.31 (1.20–1.43)				385 (18.6)	1.37 (1.22–1.54)			
None	4975 (22.1)	Reference				2724 (14.3)	Reference			
Lack of green areas										
A great deal	1252 (29.3)	1.57 (1.45–1.69)	<0.001			567 (18.4)	1.40 (0.86–1.55)	<0.001		
Somewhat	1178 (25.4)	1.29 (1.19–1.39)				619 (17.0)	1.27 (1.16–1.41)			
None	3684 (21.0)	Reference				2126 (13.9)	Reference			
Presence of animals causing a significance nuisance										
A great deal	1113 (29.0)	1.48 (1.37–1.60)	<0.001			498 (18.3)	1.34 (1.21–1.49)	<0.001		
Somewhat	1044 (24.4)	1.17 (1.08–1.27)				559 (15.8)	1.13 (1.02–1.25)			
None	3957 (21.6)	Reference				2255 (14.3)	Reference			
Number of environmental problems perceived	Mean (SD)	1.16 (1.14–1.18)	<0.001	1.29 (1.17–1.43)	<0.01	Mean (SD)	1.13 (1.11–1.51)	<0.001	1.18 (1.0.7–1.32)	0.01
	2.19 (1.89)					1.99 (1.83)				

CMD: common mental disorders; SD: Standard Deviation. OR: odds ratio; ORa: odds ratio adjusted for all sociodemographic, clinical and lifestyle habits variables and perception of environmental problems variables; 95% CI: 95% Confidence Interval; n: number of people with common mental disorders; inhab.: inhabitants; Nagelkerke’s R^2^ for women’s model: 0.25; *p*-Value for women`s model: <0.001. Nagelkerke’s R^2^ for men`s model: 0.23; *p*-Value for men’s model: <0.001.

## Supplementary Materials

The following are available online at https://www.mdpi.com/2077-0383/9/7/2199/s1, File S1: Research Data.
